# Diagnosis and follow up of patients with primary cardiac tumours: a single-centre experience of myxomas

**DOI:** 10.5830/CVJA-2010-073

**Published:** 2011-11

**Authors:** S Manduz, N Katrancioglu, O Karahan, O Yucel, MB Yilmaz

**Affiliations:** Department of Cardiovascular Surgery, Faculty of Medicine, Cumhuriyet University, Sivas, Turkey; Department of Cardiovascular Surgery, Faculty of Medicine, Cumhuriyet University, Sivas, Turkey; Department of Cardiovascular Surgery, Faculty of Medicine, Cumhuriyet University, Sivas, Turkey; Department of Cardiology, Faculty of Medicine, Cumhuriyet University, Sivas, Turkey; Department of Cardiology, Faculty of Medicine, Cumhuriyet University, Sivas, Turkey

**Keywords:** myxoma, diagnosis, surgery

## Abstract

**Objective:**

In this study, 12 patients who were diagnosed as having cardiac tumours and were operated on in the Department of Cardiovascular Surgery following referral from the Department of Cardiology were enrolled between January 1995 and October 2007.

**Methods:**

The symptoms, clinical findings, diagnostic methods, localisation of masses and surgical applications were recorded retrospectively.

**Results:**

There were 10 female (83%) and two (17%) male patients; their ages ranged from 35 to 70 years (mean 68.7 years). Twelve patients were diagnosed with myxomas, nine of which were located within the left atrium and three in the right atrium. The most common symptoms at clinical presentation were those associated with heart failure or embolisation. Diagnosis of the tumours was made by echocardiography in all patients. The masses were completely resected in eight patients and the interatrial septae were partially excised with mass resection in two patients. The defect was reconstructed with a pericardial patch in one of the patients, and primarily reconstructed in the other. We carried out debridement with mass resection in another case. Femoro–popliteal aorto–iliac thrombo-endarterectomy was performed with mass resection in a further case.

**Conclusion:**

Atrial myxomas are the most common primary cardiac tumours. They can cause valvular or inflow–outflow tract obstruction, thrombo-embolism, arrhythmias, or pericardial disorders. Most atrial myxomas are benign but due to non-specific symptoms, early diagnosis may be a challenge and they must be removed by surgical resection. Diagnosis and follow up with the collaboration of cardiology and cardiovascular surgery departments is important for meticulous care of these patients.

## Abstract

Primary cardiac tumours are rare. Their prevalence ranges from 0.0017 to 0.28% in various autopsy series, and they are up to 20 times less frequent than are secondary tumours of the heart.^1^,^2^ The prevalence of primary cardiac tumours other than benign myxomas is even lower.^1^-^3^ In 1954, Clarence Crafoord removed an intra-atrial myxoma for the first time. Echocardiography has enabled better visualisation of the cardiac structures and accurate diagnosis.^4^

Approximately 75% of sporadic myxomas occur in females. Myxomas have been reported in patients aged three to 83 years.^3^-^5^ They are rarely seen in children, accounting for only nine to 15% of all cardiac tumours from birth to adolescence.^5^

Early diagnosis and surgical removal of the tumour with decreased mortality and morbidity is related to the symptoms produced, such as tumour embolism, heart failure, mechanical valvular obstruction, and various constitutional symptoms.^6^ We retrospectively reviewed our case series with particular attention to myxomas.

## Methods

In this study, 12 patients who suffered from myxomas were diagnosed and operated on in the Departments of Cardiology and Cardiovascular Surgery, respectively, between January 1995 and October 2007. Symptoms, clinical findings, diagnostic methods, localisation of the mass and surgical application were evaluated retrospectively.

Echocardiographic examinations were performed by an experienced echocardiographer using available ultrasound equipment (GE-Vivid 4 with a 3.5 MHz transducer, Wisconsin, USA) at baseline, and as required during follow up. Pre-operative coronary angiography was performed in patients with known or suspected coronary artery disease.

## Results

There were 10 female (83%) and two (17%) male patients; their ages ranged from 35 to 70 years (mean 68.7 years). All patients were diagnosed as having myxomas on pathological verification. Nine of these myxomas were in the left atrium and three were in the right atrium. All myxomas were sporadic. The most common symptoms at clinical presentation were those associated with cardiac insufficiency or embolisation. There was haemoptysis in one patient with bronchiectasis.

**Fig. 1.**
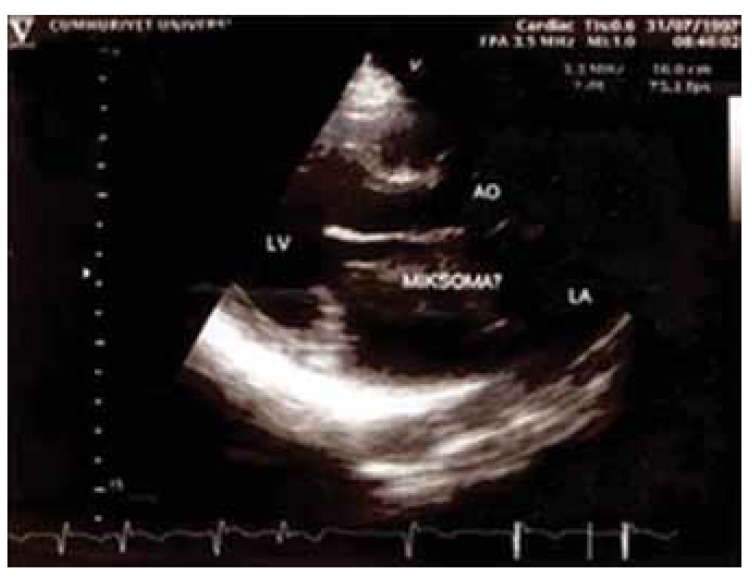
Left atrial mass (4.8 × 7.5 cm) near the interatrial septum (case 2).

**Fig. 2.**
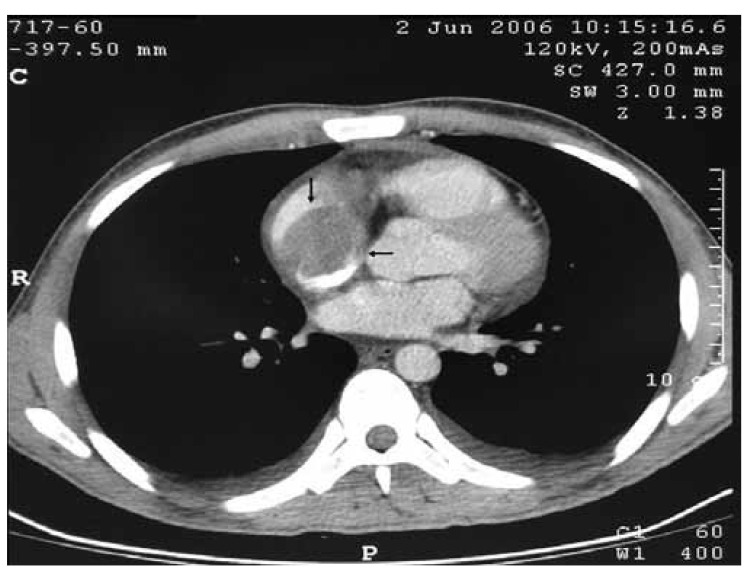
Excised mass (case 2).

Diagnosis of the tumours was made by echocardiography before surgery in all patients (Fig. 1). Computed tomography and angiocardiography were also used in some of the patients as required. The masses were completely resected in eight patients and the interatrial septae were partially excised with mass resection in two patients (Fig. 2). The defect was reconstructed with a pericardial patch in one patient and primarily reconstructed in the other. We carried out debridement with mass resection in another case. Femoro–popliteal aorto–iliac thrombo-endarterectomy was performed with mass resection in a further case and the embolectomy material was confirmed as a myxoma. Coronary artery bypass surgery was performed in a patient with coronary artery disease (Table 1).

**Table 1.** Primary Cardiac Tumours

*Case*	*Age*	*Gender*	*Final diagnosis*	*Presenting symptom*	*BP (mmHg)*	*Pulse (beats/min)*	*Rhythm*	*NYHA*	*Diagnostic methods*	*Valve disease*	*Pericardial effusion*	*Surgical procedure*	*Outcome*
1	35	F	LA myxoma	Dyspnoea	120/60	82	AF	2	Echo	2° MR	–	Tumour resection	Healing
										2° TR			
2	70	M	LA myxoma	Dyspnoea + palpitations	110/60	88	Sinus rhythm	4	Echo + CAG	3° TR	–	Tumour resection	Healing
3	65	F	LA myxoma	Dyspnoea + haemoptysis	130/70	116	AF	2	Echo + CAG	2–3° TR	–	Tumour resection	Healing
4	58	F	LA myxoma	Pain and parestesia in right leg	130/70	90	AF	2	Echo	1° MR	–	Tumour resection + pericardial patch	Healing
5	35	F	RA myxoma	Eleve fever + palpitations	140/70	96	Sinus rhythm	3	Echo	2° TR	–	Tumour resection + debridement	Healing
6	60	F	RA myxoma + CAD	Fatigue + emesis	120/60	82	Sinus rhythm	2	Echo + CAG	1° MR	–	Off-pump LIMA–LAD bypass + tumour resection	Healing
										1° TR			
7	66	F	LA myxoma	Dyspnoea + palpitations	110/70	68	AF	3	Echo + CAG	2–3° TR	+	Tumour resection + tricuspid De Vega plasty	Healing
8	62	F	RA myxoma	Dyspnoea + ankle oedema	120/70	72	Sinus rhythm	4	Echo + CAG	–	–	Tumour resection	Died
9	61	M	LA myxoma	Dyspnoea + palpitations	130/80	78	Sinus rhythm	2	Echo + CT	–	–	Tumour resection	Healing
10	57	F	LA myxoma	Pallor + pain bilaterally down extremities and enuresis	120/70	115	AF	2	Echo + abdominal CDUS	1° MR	+	Tumor resection + femoro-popliteal aorto-iliac thromboendarterctomy	Healing
										2° TR			
										Min AR			
11	60	F	LA myxoma	Dyspnoea + palpitations	120/60	70	Sinus rhythm	3	Echo	2–3°	–	Tumour resection + MVR	Healing
										MR			
										MS			
12	58	F	LA myxoma	Dyspnoea + fatigue	120/70	94	Sinus rhythm	3	Echo	2° TR	–	Tumour resection + tricuspid De Vega plasty	Healing

RA = right atrial, LA = left atrial, AF = atrial fibrillation, Echo = echocardiography, CAG = coronary angiography, CT = computed tomography, CDUS = colour Doppler ultrasonography, MR = mitral regurgitation , TR = tricuspid regurgitation, AR = aortic regurgitation, MS = mitral stenosis, LIMA–LAD = left internal mammary artery–left anterior descending artery, De Vega plasty = De Vega’s tricuspid annuloplasty, MVR = mitral valve repair.

The minimum follow-up period was 12 months and the maximum was 132 months (median, 60 months). One of the patients with myxoma died in the early postoperative period due to cerebrovascular accident.

**Fig. 3.**
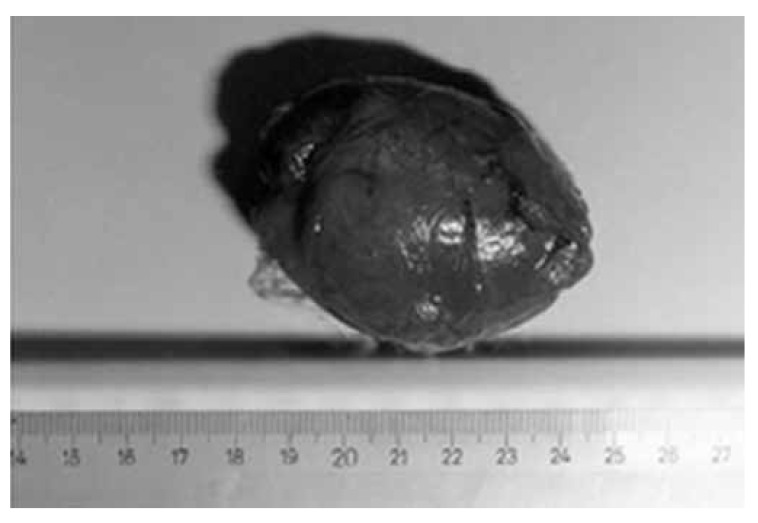
CT report: 4 × 5-cm hypo-dense lesion (myxoma) that caused a filling defect in the right atrium.

In some of the cases, pleural effusion was detected with computed tomography and chest X-ray (Fig. 3). We used abdominal and peripheral Doppler ultrasound in case 12, and found a subacute thrombus which totally obstructed the abdominal aorta, extending 3 cm in the proximal part down to the bifurcation and including 4 cm proximally in the common femoral artery. The details of the cases are presented in Table 1.

## Discussion

Cardiac myxomas most commonly occur in the third to fourth decade of life, and approximately 75% of sporadic myxomas occur in females.^2^ They frequently occur in the left atrium in 75% of cases. The right atrium is involved in 15 to 20% of cases. There is no difference in terms of frequency of involvement of the ventricles (6–8%).^2^ There were 10 female (83%) and two (17%) male patients. In addition, left atrial involvement was observed in 67% of the cases in our study, in accordance with the current literature.

Cardiac myxomas originate from primitive, multipotential mesenchymal cells present in the heart wall as embryonic remnants. Histopathologically, a myxoma is composed of an abundant acid mucopolysaccharide matrix in which polygonal cells and immature endothelial cells forming blood vessels are embedded in chronic inflammation.^5^,^7^,^8^ Masses from the 12 cases in our study were histopathologically typical of myxomas.

Myxomas cause peripheral embolisms, in which fragments of the tumour break away into the blood stream and cause clots or blockages. Systemic embolism is encountered in 30 to 45% of myxoma cases.^9^-^11^ A massive embolism can cause death. There were two cases with femoral artery embolism in our series, which were confirmed on pathological evaluation. Fortunately, in our series, no patient had a cerebral embolism, which may have been fatal.

On the other hand, aneurysms may also occur in these patients, causing symptoms associated with central nervous system involvement.^12^ Therefore a careful examination for any symptom that could possibly be associated with nervous system involvement should be an essential part of the therapeutic approach.

An important aspect of our study was that the tumour was detected in all patients by echocardiography, which was performed due to the non-specific symptoms of the patients. Coronary angiography, however, was performed in older patients who were thought to have coronary artery disease. Echocardiographic follow up was done on a routine basis in the third, sixth and twelfth months and thereafter annually in all patients. We feel that routine echocardiographic follow up may help in earlier diagnosis and timely intervention to avoid neurological sequelae.

Operative resection of the myxomas is the treatment of choice. Some authors believe that resection should be performed immediately after the diagnosis is made.^2^-^4^ We performed our operations immediately after the diagnosis.

## Conclusion

The development of diagnostic techniques and the routine practice of echocardiography have enabled us to define cardiac tumours earlier, important for both the initial diagnosis and for detecting a recurrence. Hence, mortality and morbidity were quite low in our series. Untreated benign cardiac tumours can be distressing to both patient and physician.

Treatment of myxomas is usually done by surgical removal of the tumour. Complete resection, given the low operative mortality rate that can be accomplished in experienced hands, and then follow up by serial echocardiography over five or six years to monitor for recurrences are crucial for an appropriate therapeutic approach, although late recurrences with neurological symptoms have been noted in the literature.^12^,^13^
